# Are Homeostatic States Stable? Dynamical Stability in Morphoelasticity

**DOI:** 10.1007/s11538-018-0502-7

**Published:** 2018-09-21

**Authors:** Alexander Erlich, Derek E. Moulton, Alain Goriely

**Affiliations:** 1 School of Mathematics, University of Manchester, Oxford Road, Manchester, M13 9PL UK; 2 Mathematical Institute, University of Oxford, Andrew Wiles Building, Woodstock Road, Oxford, OX2 6GG UK

**Keywords:** Nonlinear elasticity, Continuum mechanics, Biological growth, Dynamical systems, Ordinary differential equations, Discretization scheme

## Abstract

Biological growth is often driven by mechanical cues, such as changes in external pressure or tensile loading. Moreover, it is well known that many living tissues actively maintain a preferred level of mechanical internal stress, called the mechanical homeostasis. The tissue-level feedback mechanism by which changes in the local mechanical stresses affect growth is called a growth law within the theory of morphoelasticity, a theory for understanding the coupling between mechanics and geometry in growing and evolving biological materials. This coupling between growth and mechanics occurs naturally in macroscopic tubular structures, which are common in biology (e.g., arteries, plant stems, airways). We study a continuous tubular system with spatially heterogeneous residual stress via a novel discretization approach which allows us to obtain precise results about the stability of equilibrium states of the homeostasis-driven growing dynamical system. This method allows us to show explicitly that the stability of the homeostatic state depends nontrivially on the anisotropy of the growth response. The key role of anisotropy may provide a foundation for experimental testing of homeostasis-driven growth laws.

## Introduction

Biological tissues exhibit a wide range of mechanical properties and active behavior. A striking example is biological growth in response to the tissues mechanical environment. Artery walls thicken in response to increased pressure (Berry 1974; Goriely and Vandiver 2010), axons can be grown by applying tension (Lamoureux et al. 1992; Recho et al. 2016), and plant growth is driven by various mechanical cues (Goriely et al. 2008; Boudaoud 2010). The general idea underlying these phenomena is that the internal stress state is a stimulus for growth. As stress is rarely uniform, mechanically induced growth often coincides with differential growth, in which mass increase occurs non-uniformly or in an anisotropic fashion. In turn, differential growth produces residual stress, an internal stress that remains when all external loads are removed, appearing due to geometric incompatibility induced by the differential growth. Residual stress has been observed in a number of physiological tissues, such as the brain (Budday et al. 2014), the developing embryo (Beloussov and Grabovsky 2006), arteries (Fung and Liu 1989), blood vessels (Fung 1991), solid tumors (MacArthur and Please 2004), and in a wealth of examples from the plant kingdom (Goriely 2017). In many cases, residual stress has been found to serve a clear mechanical function; for instance in regulating size and mechanical properties.

Many living tissues actively grow in order to maintain a preferred level of internal residual stress, termed mechanical homeostasis. This phenomenon is characterized by growth being induced by any difference between the current stress in the tissue and the preferred homeostatic stress. Mechanically driven growth toward homeostasis poses several interesting and important questions, at the biological, mechanical, and mathematical level. For instance, what determines the homeostatic stress state? At the cellular level, the growth response may be genetically encoded, with a homeostatic state manifest by differential cellular response to mechanical stimuli. From a continuum mechanics point of view, a residually stressed configuration is typically thought of as corresponding to a deformation from an unstressed configuration; however, it is not clear that such a deformation should exist to define a homeostatic state. Connected to this is a question of compatibility: is it actually *possible* for a system to reach mechanical homeostasis? For example, the boundary of an unconstrained tissue will always be traction free, and thus, if the homeostatic stress for those boundary cells is nonzero, then the system can never completely reach homeostasis. From a dynamics point of view, there is a natural question of stability: is the homeostatic state stable, i.e., if the system is perturbed from its homeostatic equilibrium, is it able to grow in such a way to return to this state? There is also a practical issue of connecting experiment to theory: how does one quantify the homeostatic state and form of growth response?

Mathematical modeling can be of significant value in addressing such questions and in suggesting potential experimental measures to quantify the properties of homeostasis. In the simplest and most widely used form, the mathematical description involves a growth law of the form

1 $$\begin{aligned} {\mathbf {G}}^{-1}\dot{{\mathbf {G}}} = {\varvec{K}}:({\mathbf {T}}-\mathbf {T^*}). \end{aligned}$$

Here overdot represents time derivative, ${\mathbf {G}}$ is a growth tensor, characterizing the increase or decrease in mass as a local property, ${\mathbf {T}}$ is the Cauchy stress tensor, $\mathbf {T^*}$ is the preferred homeostatic stress tensor, and ${\varvec{K}}$ is a fourth-order tensor characterizing the growth response rate due to differences in current and preferred stress. Laws of the form (1), or slight variations thereof, in which growth is coupled to Cauchy stress, have been examined by a number of authors (Vandiver and Goriely 2009; Bowden et al. 2016; Ramasubramanian 2008; Taber 2008), though the most appropriate form of growth law is a much-debated issue (Taber 1995; Ambrosi et al. 2011; Jones and Chapman 2012; Goriely 2017). An alternative but related approach involves coupling growth and Eshelby stress (Ambrosi and Guana 2005) based on thermodynamical arguments (Epstein 2000; Erlich et al. 2015). Attempts to restrict the form of growth laws through thermodynamical considerations such as the Coleman–Noll procedure (Coleman and Noll 1963) have been of limited success due to the inherent thermodynamical openness and non-equilibrium nature of biological systems (Maugin 1999; Lebon et al. 2008). The integration of micro-mechanical models with tissue-level modeling has also been difficult, partly because the lack of periodicity and crystal symmetry in biological tissues makes the application of homogenization techniques difficult (Chenchiah and Shipman 2014). Growth dynamics that depend on the current stress state are inherently challenging to study analytically. Both stress and growth will tend to be spatially dependent, with stress being determined through the solution of a force balance boundary-value problem, and thus, any model will by nature involve a partial differential equation system. The situation is simplified somewhat by the *slow-growth assumption*, which states that growth occurs on a much longer timescale than the elastic timescale and hence the system is always in a quasi-static mechanical equilibrium.

In this paper, we study mechanically driven growth in the context of growing tubular structures. One motivation for a cylindrical geometry is that such structures are ubiquitous in the biological world, from plant stems (Goriely et al. 2010) to axons and airways (Moulton and Goriely 2011a, b), and exhibit diverse mechanical behavior. Working within a constrained geometry will also enable us to gain qualitative insight into the dynamics of structures with growth driven by mechanical homeostasis and to formulate a basic framework for studying the stability of a homeostatic state. Even in an idealized geometry, the full growth dynamics still consists of a set of partial differential equations, with mechanical equilibrium requiring the solution of a boundary-value problem at each time step, and a highly nonlinear growth evolution for components of the growth tensor. There is no mathematical theory, yet, that allows for such an analysis. Our approach is therefore to devise a discretization through a spatial averaging scheme that converts the system to a much more manageable initial-value problem, to which we can apply standard techniques from dynamical systems. The discretization we propose consists of defining annular layers of the tubular structure, such that growth is uniform in each layer, driven by averaged values of the stress components in a law of the form (1). While this approach enables us to study efficiently properties of the continuous (non-discretized) system as the number of layers increases, for a smaller number of layers it is also a useful model of a multilayered tube commonly found in many biological systems.

This paper is structured as follows. In Sect. 2, we discuss the general deformation and growth dynamics for a tubular structure that is homogeneous in the axial direction. In Sect. 3, we focus on a tubular system made of two layers, illustrating the main ideas of our discretization approach and illustrating the rich dynamics of this system. In Sect. 4, we generalize from two to *N* layers. Here we find a rapid convergence of behavior as the number of layers increases and investigate how the anisotropy of the growth affects the stability.

## Continuous Growth Dynamics in Cylindrical Geometry

### Kinematics

**Fig. 1:**
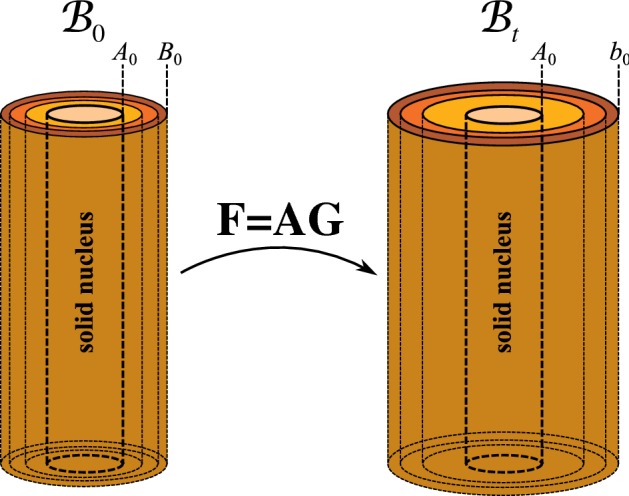
Sketch of kinematic setup

We consider a cylindrical tube, consisting of an incompressible isotropic hyperelastic material, the inner wall of which is attached to a fixed solid nucleus, with the outer wall unconstrained (see Fig. 1). We restrict to growth and deformations only in the cross section, such that the cylindrical geometry is always maintained and there is no axial strain. Moreover, we assume that there are no external forces, so that any deformation is caused purely by growth and the elastic response.

Geometrically, we work in a planar polar coordinate basis $\left\{ {\mathbf {e}}^{R},{\mathbf {e}}^{\theta }\right\}$ (the same basis vectors apply to both initial and current configurations), in which the deformation can be described by the map ${\mathbf {x}}:{\mathcal {B}}_0\rightarrow {\mathcal {B}}_t$ given by:

2 $$\begin{aligned} {\mathbf {x}}=r\left( R^{0}\right) {\mathbf {e}}^{R}\,. \end{aligned}$$

For this map, the deformation gradient is

3 $$\begin{aligned} {\mathbf {F}}=r'\left( R^{0}\right) {\mathbf {e}}^{R}\otimes {\mathbf {e}}^{R}+\frac{r}{R^{0}}{\mathbf {e}}^{\theta }\otimes {\mathbf {e}}^{\theta }. \end{aligned}$$

The elastic deformation gradient takes the form

4 $$\begin{aligned} {\mathbf {A}}=\alpha ^{R}{\mathbf {e}}^{R}\otimes {\mathbf {e}}^{R}+\alpha ^{\theta }{\mathbf {e}}^{\theta }\otimes {\mathbf {e}}^{\theta }. \end{aligned}$$

**Fig. 2:**
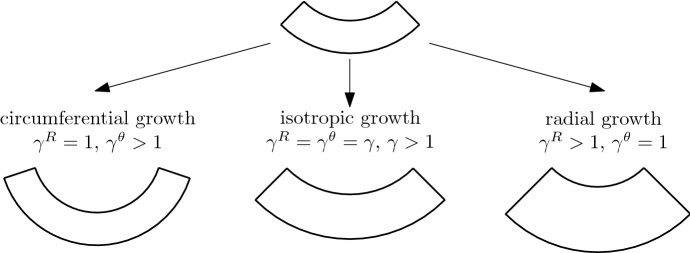
Illustration of isotropic and anisotropic growth

Incompressibility requires $\det {\mathbf {A}}=1$; we thus define $\alpha :=\alpha ^{\theta }$, so that $\alpha ^{-1}=\alpha ^{r}$. We assume a diagonal growth tensor

5 $$\begin{aligned} {\mathbf {G}}=\gamma ^{R}{\mathbf {e}}^{R}\otimes {\mathbf {e}}^{R}+\gamma ^{\theta }{\mathbf {e}}^{\theta }\otimes {\mathbf {e}}^{\theta }, \end{aligned}$$

where the difference between radial growth ($\gamma ^R>1$) and circumferential growth ($\gamma ^\theta >1$) is shown schematically in Fig. 2. In matrix form (with the basis $\left\{ {\mathbf {e}}^{R},{\mathbf {e}}^{\theta }\right\}$ implied), we have

6 $$\begin{aligned} {\mathbf {F}}=\begin{pmatrix}\frac{\mathrm {d}r}{\mathrm {d}R^{0}} &{} 0\\ 0 &{} \frac{r}{R^{0}} \end{pmatrix}\,,\qquad {\mathbf {A}}=\begin{pmatrix}\alpha ^{-1} &{} 0\\ 0 &{} \alpha \end{pmatrix}\,,\qquad {\mathbf {G}}=\begin{pmatrix}\gamma ^{R} &{} 0\\ 0 &{} \gamma ^{\theta } \end{pmatrix}\,. \end{aligned}$$

In the initial (stress-free) reference configuration ${\mathcal {B}}_{0}$, the inner cylinder wall is located at $R^{0}=A_{0}$ and the outer wall is located at $R^{0}=B_{0}$. From the morphoelastic decomposition ${\mathbf {F}}={{\mathbf {A}}}{{\mathbf {G}}}$, we find $r'=\gamma ^{R}/\alpha$ and $r/R^{0}=\alpha \gamma ^{\theta }$. By eliminating $\alpha$, we obtain

7 $$\begin{aligned} r\left( R^{0}\right) r'\left( R^{0}\right) =\gamma ^{R}\left( R^{0}\right) \gamma ^{\theta }\left( R^{0}\right) R^{0}. \end{aligned}$$

Imposing the boundary condition $r\left( A_{0}\right) =A_{0}$, due to the unmoving solid nucleus, we can integrate (7) as

8 $$\begin{aligned} r=\sqrt{A_{0}^{2}+2\int _{A_{0}}^{R^{0}}\!\!\!\gamma ^{R}({\tilde{R}})\gamma ^{\theta }({\tilde{R}}){\tilde{R}}\ \mathrm {d}{\tilde{R}}}. \end{aligned}$$

The mathematical and biological significance of our solid nucleus setup is discussed in Sect. 4.5.

### Mechanics

Given that all deformations are diagonal in the coordinate basis considered here, the Cauchy stress is also diagonal

9 $$\begin{aligned} {\mathbf {T}}=T^{RR}{\mathbf {e}}^{R}\otimes {\mathbf {e}}^{R}+T^{\theta \theta }{\mathbf {e}}^{\theta }\otimes {\mathbf {e}}^{\theta }. \end{aligned}$$

Let $W\left( \alpha ^{R},\alpha ^{\theta }\right)$ be the strain-energy density, which relates to the Cauchy stress tensor by ${\mathbf {T}} = {\mathbf {A}}W_{\mathbf {A}} - p{\mathbf {1}}$, where *p* is the Lagrange multiplier enforcing incompressibility. In components, this reads

10 $$\begin{aligned} T^{RR}=\alpha ^{R}\frac{\partial W}{{\partial \alpha }^{R}}-p\,,\qquad T^{\theta \theta }=\alpha ^{\theta }\frac{\partial W}{{\partial \alpha ^{\theta }}}-p. \end{aligned}$$

With no external loads, mechanical equilibrium requires $\text {div }{\mathbf {T}}=0$, which takes the form

11 $$\begin{aligned} \frac{\partial T^{RR}}{\partial r}=\frac{T^{\theta \theta }-T^{RR}}{r}. \end{aligned}$$

Defining ${\widehat{W}}\left( \alpha \right) :=W\left( \alpha ^{-1},\alpha \right)$, we have

12 $$\begin{aligned} T^{\theta \theta }-T^{RR}=\alpha {\widehat{W}}'(\alpha ). \end{aligned}$$

The above formulation is valid for all isotropic, incompressible material. To make progress in our analysis, we further restrict it to the simplest possible material model of a neo-Hookean quadratic strain-energy density given by:

13 $$\begin{aligned} W=\frac{\mu }{2}\left( \alpha _{R}^{2}+\alpha _{\theta }^{2}-2\right) , \end{aligned}$$

where $\mu$ in small deformations can be identified with the shear modulus of the material. In this case,

14 $$\begin{aligned} {\widehat{W}}\left( \alpha \right) =\frac{\mu }{2}\left( \alpha ^{2}+\alpha ^{-2}-2\right) \,, \end{aligned}$$

for which (11) becomes

15 $$\begin{aligned} \frac{\mathrm {d}T^{RR}}{\mathrm {d}R^{0}} =\frac{2\mu \gamma ^{R}}{R^{0}\gamma ^{\theta }}\left[ 1-\frac{\left( R^{0}\right) ^{4}\left( \gamma ^{\theta }\right) ^{4}}{r^{4}}\right] . \end{aligned}$$

Along with (15), we impose $T^{RR}\left( B_{0}\right) =0$, i.e., the outer edge is stress-free. Equations (7) and (15), along with boundary condition $T^{RR}\left( B_{0}\right) =0$, completely determine the deformation and stress state. Due to the fixed inner boundary condition, for a given growth tensor (7) can be integrated separately, i.e., the deformation is determined independently from the stress, and the radial Cauchy stress is then determined by integrating (15). Once the radial stress component $T^{RR}$ is determined, the circumferential component satisfies

16 $$\begin{aligned} T^{\theta \theta }=T^{RR}+\frac{2\mu r^{2}}{\left( R^{0}\right) ^{2}\left( \gamma ^{\theta }\right) ^{2}}\left[ 1-\frac{\left( R^{0}\right) ^{4}\left( \gamma ^{\theta }\right) ^{4}}{r^{4}}\right] \,. \end{aligned}$$

Note also that for constant $\gamma ^R$ and $\gamma ^\theta$, these integrals may be performed analytically, giving explicit expressions for the stress and deformation in terms of the growth. As we show later, the same holds when extending from one layer to multiple layers; if the growth in each layer is constant, the stress components may be written explicitly. It is this fact that we exploit below in formulating a discretized growth dynamics. This is the main motivating reason for the fixed core geometry we consider. Under different boundary conditions, the deformation and stress would be coupled, requiring for instance a root finding exercise to determine the outer radius for which the stress boundary condition is satisfied. In such a case, the framework below applies at the expense of added computational complexity.

### Growth Law

We now impose a homeostasis-driven growth law of the form (1). In the plane polar geometry, this takes the form

17 $$\begin{aligned} \begin{aligned} {\dot{\gamma }}^{R}&=\left\{ K^{RR}\left[ T^{RR}-\big (T^{RR}\big )^{*}\right] +K^{R\theta }\Big [T^{\theta \theta }-\big (T^{\theta \theta }\big )^{*}\Big ]\right\} \gamma ^{R}\,,\\ {\dot{\gamma }}^{\theta }&=\left\{ K^{\theta R}\left[ T^{RR}-\big (T^{RR}\big )^{*}\right] +K^{\theta \theta }\Big [T^{\theta \theta }-\big (T^{\theta \theta }\big )^{*}\Big ]\right\} \gamma ^{\theta }\,. \end{aligned} \end{aligned}$$

Here $K^{RR}:={\mathcal {K}}^{RRRR}$, $K^{R\theta }:={\mathcal {K}}^{RR\theta \theta }$, $K^{\theta R}:={\mathcal {K}}^{\theta \theta RR}$, $K^{\theta \theta }:={\mathcal {K}}^{\theta \theta \theta \theta }$ are the only non-vanishing components of the fourth-order tensor ${{\varvec{K}}}$, and are assumed to be constant in space and time.

### Discretization Approach

For given homeostatic stress values and components of ${{\varvec{K}}}$, the growth dynamics is fully defined, with the growth components evolving according to (17). Even in the simplified cylindrical geometry, this comprises a system of nonlinear partial differential equations. Moreover, viewing the dynamics as a discrete process is still complicated by the fact that at each time step updating the growth requires knowing the stress components, which requires integration of (15), which requires integration of (7), which cannot be done analytically for general spatially dependent $\gamma ^R$ and $\gamma ^\theta$.

However, as stated above, for constant $\gamma ^R$ and $\gamma ^\theta$, the integrals determining stress may be computed analytically. This suggests a discretization process whereby the annular domain is divided into discrete layers, each with constant growth, and such that the growth in each layer evolves according to averaged values of the stress. In this way, analytical expressions may be determined for both the stress and the average stress, and hence, the dynamics is reduced to a set of ordinary differential equations for the growth components. The inhomogeneity of the full model is replaced by a piecewise homogeneous model. This preserves the key idea of inhomogeneity (allowing, for instance, circumferential growth to be higher near the nucleus than away from it), but is more analytically tractable and allows for precise statements about the long-term dynamics, stability, and qualitative investigation such as the influence of radial versus circumferential stress to the growth dynamics.

## Growth Dynamics for 2-Layer System

### Kinematics

**Fig. 3:**
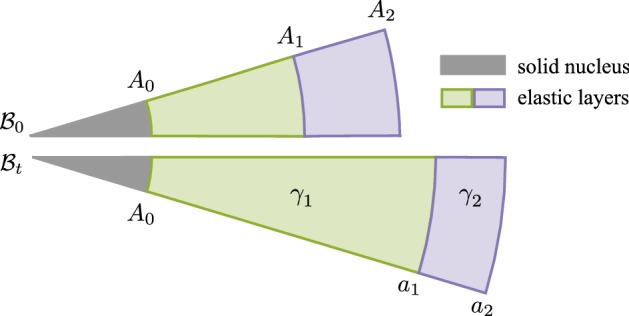
Kinematic setup for the two-layer system. The innermost layer is attached to an unmoving nucleus ($a_{0}=A_{0}$) and the boundary condition at the outer layer is no pressure $T^{RR}\left( A_{2}\right) =0$

We first consider two elastic layers attached to a solid nucleus and in perfect mechanical contact at their interface. In the initial reference configuration ${\mathcal {B}}_{0}$, the inner wall has the radial coordinate $R^{0}=A_{0}$, the middle wall at $R^{0}=A_{1}$ and the outer wall at $R^{0}=A_{2}$. In the current configuration ${\mathcal {B}}_{t}$, the same material points have coordinates that are $r\left( A_{0}\right) =A_{0}$, $r\left( A_{1}\right) =a_{1}$ and $r\left( A_{2}\right) =a_{2}$ (see Fig. 3).

We impose that in the reference configuration the two annular layers enclose the same area $\pi \varDelta ^{2}$ . The initial reference radii of the two rings thus satisfy

18 $$\begin{aligned} \varDelta ^{2}=A_{2}^{2}-A_{1}^{2}=A_{1}^{2}-A_{0}^{2}\,. \end{aligned}$$

The deformation follows the same equations formulated in Sect. 2.1, but with piecewise homogeneous growth

19 $$\begin{aligned} \gamma \left( R^{0}\right) ={\left\{ \begin{array}{ll} \gamma _{1} &{} \text {if }A_{0}\le R^{0}\le A_{1},\\ \gamma _{2} &{} \text {if }A_{1}<R^{0}\le A_{2}\,. \end{array}\right. } \end{aligned}$$

where $\gamma _1$ and $\gamma _2$ are constant. Note that our convention is to use subscript to denote different layers and superscripts for the coordinate basis index. Here, we have imposed isotropic growth, i.e., $\gamma _{1}^{R}=\gamma _{1}^{\theta }=\gamma _{1}$ and $\gamma _{2}^{R}=\gamma _{2}^{\theta }=\gamma _{2}$. The same ideas apply for anisotropic growth, but this simplification reduces the dynamics to a 2D phase space for $\gamma _1$, $\gamma _2$. In principle, one could also have piecewise material properties and piecewise ${\varvec{K}}$ values; however, our objective is to consider the dynamics in a reduced parameter space, hence the only distinction between the layers is the different growth rates.

The deformation in each layer comes from integrating (8), subject to $r\left( A_{0}\right) =A_{0}$ and $r\left( A_{1}\right) =a_{1}$. We obtain

20 $$\begin{aligned} r\left( R^{0}\right) ={\left\{ \begin{array}{ll} r_{1}\left( R^{0}\right) :=\sqrt{A_{0}^{2}+\gamma _{1}^{2}\left[ \left( R^{0}\right) ^{2}-A_{0}^{2}\right] } &{} \text {if }A_{0}\le R^{0}\le A_{1}\,,\\ r_{2}\left( R^{0}\right) :=\sqrt{A_{0}^{2}+\gamma _{1}^{2}\varDelta ^{2}+\gamma _{2}^{2}\left[ \left( R^{0}\right) ^{2}-A_{1}^{2}\right] } &{} \text {if }A_{1}< R^{0}\le A_{2}\,. \end{array}\right. } \end{aligned}$$

Note that at $R^{0}=A_{1}$, *r* is continuous but not differentiable.

### Mechanics

The stress balance (15) determines the radial stress as

21 $$\begin{aligned}&T^{RR}\left( R^{0}\right) \nonumber \\&\quad ={\left\{ \begin{array}{ll} T_{1}^{RR}\left( R^{0}\right) :={T_{2}^{RR}\left( A_{1}\right) }+\mu \displaystyle \int _{A_{1}}^{R^{0}}\frac{2}{{\tilde{R}}}\left( 1-\frac{{\tilde{R}}^{4}\gamma _{1}^{4}}{r_{1}^{4}}\right) \mathrm {d}{\tilde{R}}, &{} R^{0}\in [A_{0},A_{1}],\\ T_{2}^{RR}\left( R^{0}\right) :={\underbrace{T^{RR}\left( A_{2}\right) }_{0}}+\mu \displaystyle \int _{A_{2}}^{R^{0}}\frac{2}{{\tilde{R}}}\left( 1-\frac{{\tilde{R}}^{4}\gamma _{2}^{4}}{r_{2}^{4}}\right) \mathrm {d}{\tilde{R}}, &{} R^{0}\in [A_{1},A_{2}]. \end{array}\right. } \end{aligned}$$

From (16), we then obtain the circumferential stress $T^{\theta \theta }\left( R^{0}\right)$:

22 $$\begin{aligned}&T^{\theta \theta }\left( R^{0}\right) =\\ \nonumber&{\left\{ \begin{array}{ll} T_{1}^{\theta \theta }\left( R^{0}\right) :=T_{1}^{RR}\left( R^{0}\right) +\mu \frac{2r_{1}^{2}}{\gamma _{1}^{2}\left( R^{0}\right) ^{2}}\left[ 1{-\frac{\left( R^{0}\right) ^{4}\gamma _{1}^{4}}{r_{1}^{4}}}\right] , &{} R^{0}\in [A_{0},A_{1}],\\ T_{2}^{\theta \theta }\left( R^{0}\right) :=T_{2}^{RR}\left( R^{0}\right) +\mu \frac{2r_{2}^{2}}{\gamma _{2}^{2}\left( R^{0}\right) ^{2}}\left[ 1-\frac{\left( R^{0}\right) ^{4}\gamma _{2}^{4}}{r_{2}^{4}}\right] , &{} R^{0}\in [A_{1},A_{2}]. \end{array}\right. } \end{aligned}$$

**Fig. 4:**
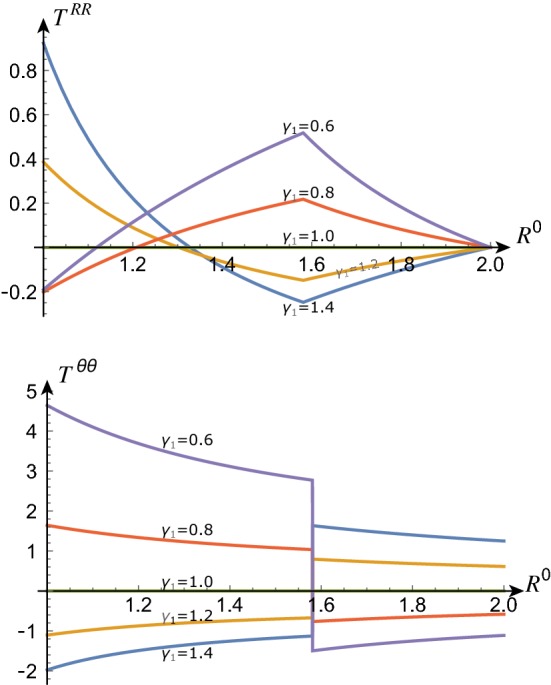
Radial (top) and circumferential (bottom) components of Cauchy stress for $A_{0}=1$, $A_{1}=\sqrt{5/2}$, $A_{2}=2$, $\varDelta =\sqrt{5/2}$, $\mu =1$, $\gamma _{2}=1$ and $\gamma _{1}$ as indicated

The expressions $T_{1}^{RR}$ and $T_{2}^{RR}$ as well as $T_{1}^{\theta \theta }$ and $T_{2}^{\theta \theta }$ can be determined analytically as functions of $A_{0}$, $A_{1}$, $A_{2}$, $\mu$, $\gamma _{1}$ and $\gamma _{2}$, though the exact expressions are long and have been suppressed here.

We note that the radial stress component is continuous at the interface between layers. This can be seen by evaluating $T^{RR}_2$ at $A_1$ in (21), which gives $T^{RR}_1(A_1)=T^{RR}_2(A_1)$. The circumferential stress, however, is discontinuous at the interface unless the growth rates of adjacent layers are equal, $\gamma _1=\gamma _2$. See also Fig. 4.

Sample stress profiles for varying values of $\gamma _1$ (with $\gamma _2=1$) are given in Fig. 4. With $\gamma _1>1$, the inner layer grows uniformly; hence, its reference state is a uniformly expanded annulus; however, it is constrained by attachment to the core and to the ungrowing outer layer. Thus the inside of the inner layer is in radial tension (the inner edge is “stretched” radially to match the core), the outside is in radial compression, and the entire layer is in compression in the hoop direction. The outer layer, on the other hand, is forced to expand circumferentially to accommodate the growing inner layer and is in circumferential compression; this is balanced by a compression in the radial direction. The inverse effect occurs with $\gamma _1<1$.

### Growth Law

We define the average stresses $\overline{T_{1}}$ and $\overline{T_{2}}$, for both radial and circumferential stress components, as

23 $$\begin{aligned} \overline{T_{1}}=\frac{2}{\varDelta ^{2}}\int _{A_{0}}^{A_{1}}T_{1}\left( {\tilde{R}}\right) {\tilde{R}}\mathrm {d}{\tilde{R}}\,,\qquad \overline{T_{2}}=\frac{2}{\varDelta ^{2}}\int _{A_{1}}^{A_{2}}T_{2}\left( {\tilde{R}}\right) {\tilde{R}}\mathrm {d}{\tilde{R}}. \end{aligned}$$

Our approach is to modify the growth dynamics so that the (constant) growth in each layer evolves according to the averaged stress values. That is, we study the system

24 $$\begin{aligned} \begin{aligned} {\dot{\gamma }}_{1}&=\gamma _{1}\left\{ K^{RR}\left[ \overline{T_{1}^{RR}}-\big (T_{1}^{RR}\big )^{*}\right] +K^{\theta \theta }\left[ \overline{T_{1}^{\theta \theta }}-\big (T_{1}^{\theta \theta }\big )^{*}\right] \right\} \,\\ {\dot{\gamma }}_{2}&=\gamma _{2}\left\{ K^{RR}\left[ \overline{T_{2}^{RR}}-\big (T_{2}^{RR}\big )^{*}\right] +K^{\theta \theta }\left[ \overline{T_{2}^{\theta \theta }}-\big (T_{2}^{\theta \theta }\big )^{*}\right] \right\} . \end{aligned} \end{aligned}$$

Note that the isotropic growth enforces $K^{RR}=K^{\theta R}$ and $K^{\theta \theta }=K^{R\theta }$; hence, there are only two (rather than four) growth rate constants $K^{RR}$ and $K^{\theta \theta }$. To further reduce the parameter space, we make the additional assumption that the homeostatic stress values are equivalent in layers 1 and 2, that is

25 $$\begin{aligned} \big (T^{RR}\big )^{*}:=\big (T_{1}^{RR}\big )^{*}=\big (T_{2}^{RR}\big )^{*}\qquad \text {and}\qquad \big (T^{\theta \theta }\big )^{*}:=\big (T_{1}^{\theta \theta }\big )^{*}=\big (T_{2}^{\theta \theta }\big )^{*}\,. \end{aligned}$$

We emphasize that while $\overline{T_{i}^{RR}}$ and $\overline{T_{i}^{\theta \theta }}$ for $i=1,2$ are averages over actual stresses according to (23), the homeostatic values $\left( T_{i}^{RR}\right) ^{*}$ and $\left( T_{i}^{\theta \theta }\right) ^{*}$ for $i=1,2$ are prescribed values that may, but need not, correspond to averages of physically realizable stresses.

To facilitate the analysis, we rescale all stress quantities by a characteristic value $\sigma$, e.g., ${\hat{T}}^{RR}=T^{RR}/\sigma$, and rescale time as ${\hat{t}}=t\sigma K^{\theta \theta }$. We also introduce

26 $$\begin{aligned} {\tilde{K}}:=K^{RR}/K^{\theta \theta }\qquad \text {and}\qquad {\hat{T}}^{*}:={\tilde{K}}\left( {\hat{T}}^{RR}\right) ^{*}+\left( {\hat{T}}^{\theta \theta }\right) ^{*}\,. \end{aligned}$$

The parameter ${\tilde{K}}$ is a measure of anisotropy of the mechanical feedback, i.e., a weighting of the contribution of radial versus circumferential stress to the (isotropic) growth response. The rescaled growth law is then

27 $$\begin{aligned} \begin{aligned} {\dot{\gamma }}_{1}&=\gamma _{1}\left[ {\tilde{K}}\overline{T_{1}^{RR}}+\overline{T_{1}^{\theta \theta }}-T^{*}\right] ,\\ {\dot{\gamma }}_{2}&=\gamma _{2}\left[ {\tilde{K}}\overline{T_{2}^{RR}}+\overline{T_{2}^{\theta \theta }}-T^{*}\right] . \end{aligned} \end{aligned}$$

Here we have re-defined the overdot as derivative with respect to the rescaled time, and we have dropped all hats for notational convenience. Note that all stress averages depend nonlinearly on $\gamma _{1}$ and $\gamma _{2}$, but not on the spatial coordinate $R^{0}$, which has been integrated out.

### Stability Analysis

To investigate the behavior of the growth dynamics, we can now apply standard techniques of dynamical systems to (27); that is we seek equilibria satisfying ${\dot{\gamma }}_{1}=0$ and ${\dot{\gamma }}_{2}=0$ and compute their stability. Let $\left\{ \gamma _{1}^{\text {eq}},\gamma _{2}^{\text {eq}}\right\}$ denote an equilibrium state. The nonlinear nature of the dependence of $\overline{T_{1}^{RR}}$, $\overline{T_{2}^{RR}}$, $\overline{T_{1}^{\theta \theta }}$ and $\overline{T_{2}^{\theta \theta }}$ on $\gamma _{1}$, $\gamma _{2}$ makes it difficult to compute analytically the number and location of equilibrium states as a function of the parameters ${\tilde{K}}$ and $T^{*}$ and we shall use numerical methods to this end.

For a given equilibrium state, we then perform a linear stability analysis. Let $0<\varepsilon \ll 1$ and expand as

28 $$\begin{aligned} \begin{aligned} \gamma _{1}&=\gamma _{1}^{\text {eq}}+\varepsilon {\overline{\gamma }}_{1}+{\mathcal {O}}\left( \varepsilon ^{2}\right) ,\\ \gamma _{2}&=\gamma _{2}^{\text {eq}}+\varepsilon {\overline{\gamma }}_{2}+{\mathcal {O}}\left( \varepsilon ^{2}\right) . \end{aligned} \end{aligned}$$

Introducing $\varvec{\gamma }=\left( \gamma _{1},\gamma _{2}\right)$ to describe the state of the system (27), its linearly expanded version (to order $\varepsilon$) takes the form

29 $$\begin{aligned} \dot{\overline{\varvec{\gamma }}}={\mathbf {J}}\varvec{{\overline{\gamma }}}, \end{aligned}$$

where the Jacobian matrix has entries

30 $$\begin{aligned} J_{ij}=\left[ \frac{\partial {\dot{\gamma }}_{i}}{\partial \gamma _{j}}\right] _{\varvec{\gamma }=\varvec{\gamma }^{\text {eq}}}. \end{aligned}$$

Stability is determined in the usual way by the form of eigenvalues of ${\mathbf {J}}$, which are the roots of the characteristic equation

31 $$\begin{aligned} 0=(J_{11}-\lambda )(J_{22}-\lambda )-J_{12}J_{21}. \end{aligned}$$

**Fig. 5:**
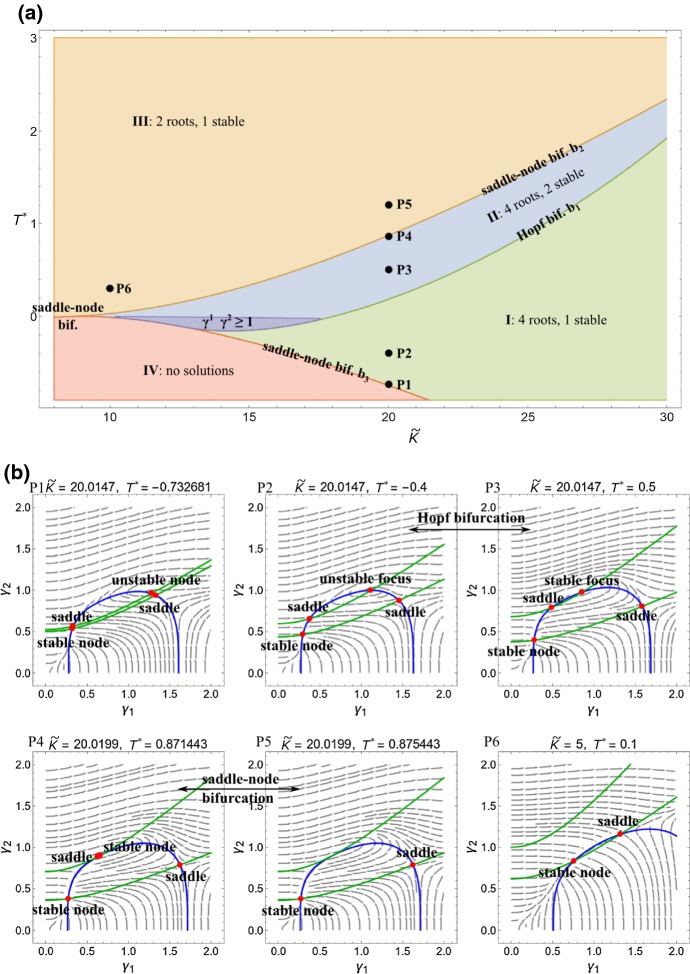
**a** Bifurcation diagram for two layered actively growing piecewise homogeneous system. **b** Equilibrium states and their dynamical characterization. Parameter values were $A_0=1$, $A_1=1.562$, $A_2=1.970$

### Bifurcation Diagram

The number of equilibrium states and their stability depend on the values of ${\tilde{K}}$ and $T^*$. In Fig. 5a, we present a phase diagram that shows four regions with distinct dynamical behavior. These can be summarized as follows:

- **Region I** has four equilibrium states, of which one is a stable node, two are saddles, and the fourth is either an unstable node or an unstable focus.
- **Region II** has four equilibrium states: two are saddles and the other two are either stable nodes or a stable focus and stable node. A Hopf bifurcation at the interface of Regions I & II transforms the unstable focus into a stable focus.
- **Region III **has two equilibrium states, one of which is a stable node, the other a saddle node. At the interface between Regions II and III, a saddle node bifurcation occurs that annihilates the stable node and saddle node in Region II.
- **Region IV** has no equilibrium states.

In Fig. 5b, we show phase portraits for the selected points P1–P5. Nullclines are plotted as blue and green curves, illustrating the appearance and disappearance of equilibrium states as categorized above.

As is evident in Fig. 5, there is a wealth of possible dynamical behavior exhibited in this system. That an idealized two-layer model with isotropic growth and equivalent homeostatic values in each layer has such a rich structure highlights a more generic complex nature of mechanically driven growth. Our intent is not to fully categorize the behavior; rather this system should be seen as a paradigm to illustrate complex dynamics. Nevertheless, several observations are in order.

One observation from the phase portraits in Fig. 5b is that unbounded growth is not only possible but “common,” at least in the sense that many parameter choices and initial conditions lead to trajectories for which $\gamma _i\rightarrow \infty$. Perhaps the most natural initial condition is to set $\gamma _1=\gamma _2=1$, which corresponds to letting the system evolve from an initial state with no growth. Examining the trajectories in Fig. 5b shows that points P1 and P2 would not evolve toward the single stable state, but rather would grow without bound.

Another point of interest is that while regions I, II and III contain stable equilibria, the stable states in Regions I and III satisfy $\gamma _{1}^{\text {eq}}\gamma _{2}^{\text {eq}}<1$. These are equilibria for which one of the layers has lost mass (at least one of the $\gamma _i<1$). Growth in both layers requires both $\gamma _i>1$, and we find that such an equilibrium only exists in a small subset of Region II, shaded dark blue in Fig. 5. We further see that $T^*<0$ in the dark blue region, and ${\tilde{K}}$ approximately in the range 10–17. This implies that in order for a stable equilibrium to exist where both layers have grown, the homeostatic stress must be compressive in one or both components, and the system must respond more strongly to radial than to circumferential stress.

*Admissible Versus Inadmissible Homeostatic Values.* In Fig. 5, we imposed the homeostatic stress $T^*$ to be equal in each layer. Moreover, $T^*$ could take any value and thus had no direct correspondence to a physically realizable stress state. We now define an *admissible homeostatic value* as the average over a stress field that can be physically realized with the given geometry and boundary conditions. Such an admissible homeostatic stress state derives from a homeostatic growth, i.e., a given growth field $\varvec{\gamma }^{*}=\left( \gamma _{1}^{*},\gamma _{2}^{*}\right) ^\mathrm{T}$ defines a spatially dependent stress, and averaging according to (23) then gives admissible values for the homeostatic stress:

32 $$\begin{aligned} \overline{T_{i}^{RR}}\left( \varvec{\gamma }^{*}\right) \qquad \text {and}\qquad \overline{T_{i}^{\theta \theta }}\left( \varvec{\gamma }^{*}\right) ,\qquad i=1,2\,. \end{aligned}$$

An *inadmissible homeostatic value* is one that cannot be expressed as an average over an actual stress, i.e., there exists no $\varvec{\gamma }^{*}$ defining $\overline{{\mathbf {T}}}^{*}$.

**Fig. 6:**
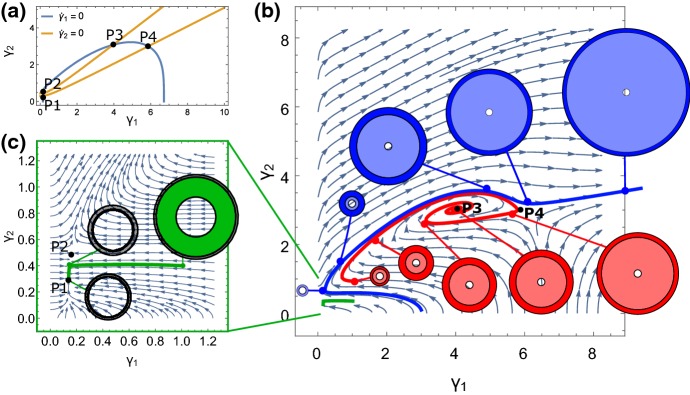
Trajectories and layer sizes for highly anisotropic growth law with admissible homeostatic state. **a** Contours for ${\dot{\gamma }}_{1}=0$ and ${\dot{\gamma }}_{2}=0$ for the system 6. As can be confirmed from the stream plots **b**, **c**, there is one stable spiral, two saddles, and one stable node. The saddle point P4 in (**b**) is the homeostatic equilibrium $\left( \gamma _{1}^{*},\gamma _{2}^{*}\right)$. Parameters: $\mu =2$, $\varDelta =\sqrt{3}$ ($A_{0}=1$, $A_{2}=\sqrt{7}$). ${\tilde{K}}=23.5$. Homeostatic growth: $\gamma _{1}^{*}=5.867$, $\gamma _{2}^{*}=3$

*Growth Law with Admissible Homeostatic Values.* To conclude our analysis of the two-layer system, we return to the same growth law, but for admissible homeostatic values. Due to the spatial inhomogeneity of the stress profile in the two-layer cylinder (see for instance Fig. 4), it is not possible to have equal homeostatic values in each layer 1 and 2. The growth law with admissible homeostatic values reads

33 $$\begin{aligned} \begin{aligned} {\dot{\gamma }}_{1}&=\gamma _{1}\left\{ {\tilde{K}}\left[ \overline{T_{1}^{RR}}\left( \varvec{\gamma }\right) -\overline{T_{1}^{RR}}\left( \varvec{\gamma }^{*}\right) \right] +\left[ \overline{T_{1}^{\theta \theta }}\left( \varvec{\gamma }\right) -\overline{T_{1}^{\theta \theta }}\left( \varvec{\gamma }^{*}\right) \right] \right\} ,\\ {\dot{\gamma }}_{2}&=\gamma _{2}\left\{ {\tilde{K}}\left[ \overline{T_{2}^{RR}}\left( \varvec{\gamma }\right) -\overline{T_{2}^{RR}}\left( \varvec{\gamma }^{*}\right) \right] +\left[ \overline{T_{2}^{\theta \theta }}\left( \varvec{\gamma }\right) -\overline{T_{2}^{\theta \theta }}\left( \varvec{\gamma }^{*}\right) \right] \right\} \,. \end{aligned} \end{aligned}$$

The phase space for this system is now inherently three dimensional, as the homeostatic stress values are defined by the two choices $\gamma _i^*$ as opposed to the single value $T^*$. Here we restrict our analysis to a single example, with $\gamma _{1}^{*}=5.867$, $\gamma _{2}^{*}=3$, and ${\tilde{K}}=23.5$, thus representing a preferred state defined by significant growth in each layer, and with strongly anisotropic growth dynamics due to the large value of ${\tilde{K}}$. The dynamics are presented in Fig. 6. The contour plot in Fig. 6a shows that there are in total four equilibrium states. The streamlines and trajectory plots in Fig. 6b, c reveal that the equilibria consist of a stable spiral, two saddles, and one stable node. It is interesting to note that P4, which is the equilibrium state at which both $\gamma _{i}^{\text {eq}}=\gamma _i^*$, is unstable; that is, the system does not remain at the equilibrium state through which the homeostatic values were defined.

Included in Fig. 6b are three sample trajectories, with the size of each layer shown at different times, and illustrative of the variety of dynamical behavior. The green trajectory quickly settles to a stable state marked by significant resorption (both $\gamma _i<1$); the blue and red trajectories sit outside the basin of attraction of P1 and show an initial period of resorption followed by significant growth. The red trajectory is in the basin of attraction of the stable focus and thus oscillates between growth and decay as it approaches the stable point at P3, while the blue trajectory, just outside the basin of attraction, ultimately grows without bound, never reaching an equilibrium state.

## Growth of Discrete *N* Layer System

Next, we generalize the dynamical system of the previous section from two to *N* layers where growth and stresses are constant throughout each layer. If *N* is sufficiently large, a system of *N* layers can be used as a suitable spatial discretization of a continuous growth profile on which precise statements can be obtained. In this case, we can generalize Eq. (33) to *N* coupled ODEs. We will analyze the stability of this system near a homeostatic equilibrium and show to what extent the results obtained for $N=2$ remain unchanged as the discretization is refined (*N* increases), which informs the stability of the continuous ($N\rightarrow \infty$) system.

A major difference compared to the two-layer model is the method to obtain homeostatic values. Previously, homeostatic values were prescribed via the homeostatic growth values $\gamma _{1}^{*}$, $\gamma _{2}^{*}$. In the present model, homeostatic values are obtained by assuming the existence of a prescribed continuous homeostatic growth profile $\gamma ^{*}\left( R^{0}\right)$. The homeostatic values $\left\{ \gamma _{i}^{*}\right\}$ are then obtained through local averaging of the prescribed profile $\gamma ^{*}\left( R^{0}\right)$ over an interval by generalizing Eq. (23). These values are admissible by construction.

Since growth is taken as constant in each layer, the stresses can be determined fully analytically and a stability analysis can then be performed. The stability analysis will inform under which conditions the dynamical system will either relax to a homeostatic state after a small perturbation or lead to an instability.

### Kinematics

**Fig. 7:**
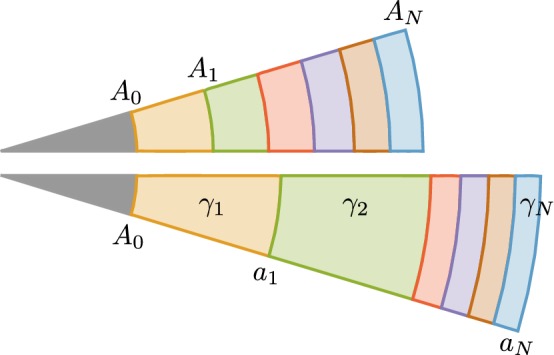
Kinematic setup for an isotropically growing *N* layered system. Note that the discretization is chosen such that the areas of each layer are equal

We consider *N* perfectly connected annuli, separated by $N+1$ interfaces, which in the initial reference configuration have the radial coordinate values $\left\{ A_{0},A_{1},\ldots ,A_{N}\right\}$ as sketched in Fig. 7. The *K*-th annulus is defined by $A_{K-1}\le R\le A_{K}$ for $K\in \left\{ 1,\ldots ,N\right\}$. We choose a particular discretization so that the area between layers, $\pi \varDelta ^{2}$, is constant:

34 $$\begin{aligned} A_{K}^{2}-A_{K-1}^{2}:=\varDelta ^{2}=\text {const.} \end{aligned}$$

We can write $A_{K}$ explicitly as

35 $$\begin{aligned} A_{K}^{2}=A_{0}^{2}+K\varDelta ^{2}\,. \end{aligned}$$

Given a continuous curve $\gamma \left( R^{0}\right)$, we define the piecewise constant growth profile by taking the average

36 $$\begin{aligned} \gamma _{K}:=\overline{\gamma \left( R^{0}\right) }=\frac{2}{\varDelta ^{2}}\int _{A_{K-1}}^{A_{K}}\gamma \left( {\tilde{R}}\right) {\tilde{R}}\mathrm {d}{\tilde{R}},\quad K=1,\ldots ,N. \end{aligned}$$

**Fig. 8:**
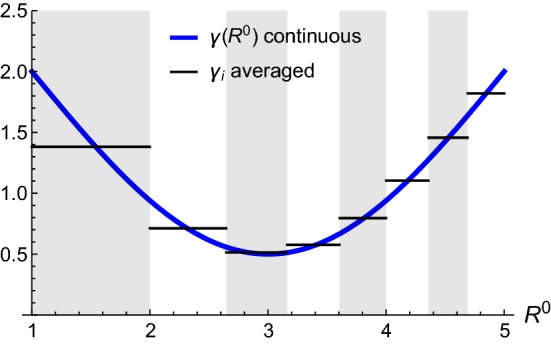
Growth $\gamma$ continuous versus averaged. The continuous curve (37) is plotted in blue, and the average over a particular discretization according to (36) is shown by a solid piecewise constant black curve ($N=8$ with $A_{0}=1$, $A_{N}=5$ and $\varDelta =\sqrt{3}$)

The growth value $\gamma _{K}$ is constant for all *K*. We demonstrate the construction of the discrete profile $\left\{ \gamma _{K}\right\}$ from the continuous profile $\gamma \left( R^{0}\right)$ in Fig. 8, in which we consider as an example the continuous function

37 $$\begin{aligned} \gamma \left( R^{0}\right) =2-\frac{3}{2}\sin \left( \pi \frac{R^{0}-A_{0}}{A_{N}-A_{0}}\right) . \end{aligned}$$

Once $\left\{ \gamma _{K}\right\}$ are obtained, we compute the radial map $r_{K}\left( R^{0}\right)$ from the discrete profile $\left\{ \gamma _{K}\right\}$. Note that while $\gamma _{K}$ is a constant throughout the *K*-th layer, the radial map $r_{K}$ is a function of the radial coordinate $R^{0}$:

38 $$\begin{aligned} r_{K}^{2}\left( R^{0}\right) =r_{K-1}^{2}\left( A_{K-1}\right) +\gamma _{K}^{2}\left[ \left( R^{0}\right) ^{2}-A_{K-1}^{2}\right] ,\qquad r_{0}^{2}\left( R^{0}\right) =A_{0}^{2}. \end{aligned}$$

Explicitly, this implies

39 $$\begin{aligned} r_{K}^{2}\left( R^{0}\right)&=A_{0}^{2}+\left( \varDelta ^{2}\sum _{i=1}^{K-1}\gamma _{i}^{2}\right) +\gamma _{K}^{2}\left[ \left( R^{0}\right) ^{2}-A_{K-1}^{2}\right] . \end{aligned}$$

**Fig. 9:**
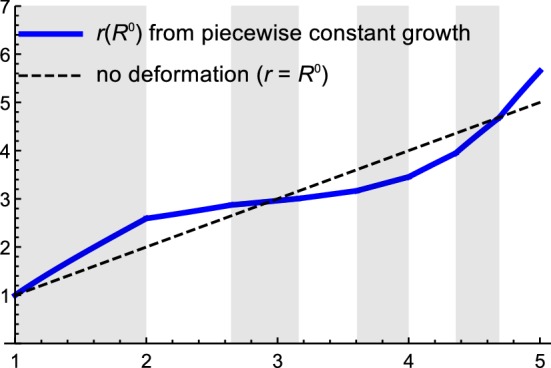
Radial function $r_{K}\left( R^{0}\right)$ for the case of discrete growth $\gamma _{i}$, computed according to (39). The dashed line represents the case of no deformation $r=R^{0}$; everything below the dashed line is resorption (“shrinking”), everything above this line is growth (Parameters as in Fig. 8)

Notice that the recursive expression (38) and the explicit expression (39) are consistent with the requirement

40 $$\begin{aligned} r_{K-1}\left( A_{K-1}\right) =r_{K}\left( A_{K-1}\right) , \end{aligned}$$

which means that *r* is continuous at each interface $A_{K-1}$ (Fig. 9).

### Mechanics

*Stress Components.* In the continuous version, the radial stress $T^{RR}$ is obtained from (15). The discrete version reads

41 $$\begin{aligned} \frac{\partial T_{K}^{RR}}{\partial R^{0}}=\frac{2\mu }{R^{0}}\left[ 1-\frac{\gamma _{K}^{4}\left( R^{0}\right) ^{4}}{r_{K}^{4}\left( R^{0}\right) }\right] ,\qquad T_{N}^{RR}\left( A_{N}\right) =0\,. \end{aligned}$$

Traction continuity at the interfaces implies

42 $$\begin{aligned} T_{K}^{RR}\left( A_{K}\right) =T_{K+1}^{RR}\left( A_{K}\right) \,. \end{aligned}$$

We define $\tau ^{RR}\left( R^{0}\right)$ as the indefinite integral over the right hand side of (41) (dropping the integration constant),

43 $$\begin{aligned} \tau ^{RR}\left( R^{0}\right) :=-\mu \frac{r_{K-1}^{2}\left( A_{K-1}\right) -A_{K-1}^{2}\gamma _{K}^{2}}{r_{K}^{2}\left( R^{0}\right) }-\mu \log \left[ \frac{r_{K}^{2}\left( R^{0}\right) }{\left( R^{0}\right) ^{2}}\right] , \end{aligned}$$

from which, we express the radial stress in the *K*-th layer as

44 $$\begin{aligned} T_{K}^{RR}\left( R^{0}\right) =\tau _{K}^{RR}\left( R^{0}\right) -\tau _{N}^{RR}\left( A_{N}\right) +\sum _{i=K}^{N-1}\mu \frac{A_{i}^{2}\left( \gamma _{i+1}^{2}-\gamma _{i}^{2}\right) }{r_{i}^{2}\left( A_{i}\right) }. \end{aligned}$$

The circumferential stress $T^{\theta \theta }$ is related to the radial stress $T^{RR}$ through (16). The discrete version of the relationship between $T^{RR}$ and $T^{\theta \theta }$ is given by

45 $$\begin{aligned} T_{K}^{\theta \theta }\left( R^{0}\right) =T_{K}^{RR}\left( R^{0}\right) +\kappa _{K}\left( R^{0}\right) , \end{aligned}$$

where

46 $$\begin{aligned} \kappa _{K}\left( R^{0}\right) :=\frac{2\mu r_{K}^{2}\left( R^{0}\right) }{\gamma _{K}^{2}\left( R^{0}\right) ^{2}}\left( 1-\frac{\gamma _{K}^{4}\left( R^{0}\right) ^{4}}{r_{K}^{4}}\right) . \end{aligned}$$

**Fig. 10:**
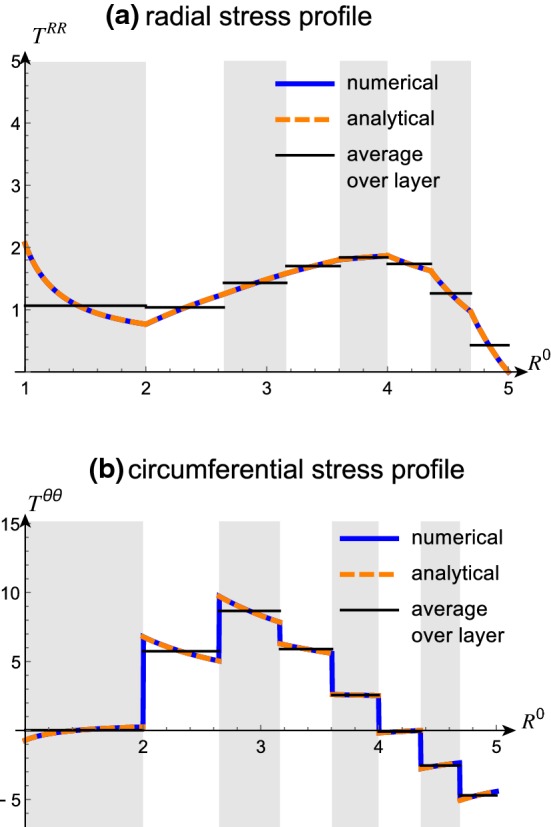
Stress profile and stress averages for the growth profile (37). **a** Radial stress profile $T^{RR}$ and average stress profile $\overline{T^{RR}}$. The analytical curve was obtained from (44) and the numerical curve (for validation) was obtained from (15). In both the numerical and analytical case, the piecewise growth profile $\gamma _{i}$ according to (36) was used. The average stress was computed according to (47) with the same growth profile as the other curves. **b** Circumferential stress profile $T^{\theta \theta }$ and average stress profile $\overline{T^{\theta \theta }}$. The analytical curve was obtained from (45) and the numerical curve (for validation) was obtained from (16). The average stress was computed according to 49. All other parameters are as in Fig. 8, with Young’s modulus $\mu =1$

Stress profiles corresponding to the growth law (37) are depicted in Fig. 10a (radial) and Fig. 10b (circumferential).

*Average Stress.* As in the two-layer case, average values for the radial and circumferential stress can be computed exactly. The average radial stress in the *K*-th layer $\overline{T_{K}^{RR}}$ is

47 $$\begin{aligned} \overline{T_{K}^{RR}}=-\tau _{N}^{RR}\left( A_{N}\right) +\sum _{i=K}^{N-1}\mu \frac{A_{i}^{2}\left( \gamma _{i+1}^{2}-\gamma _{i}^{2}\right) }{r_{i}^{2}\left( A_{i}\right) }+\frac{2}{\varDelta ^{2}}\left[ \nu _{K}^{rr}\left( A_{K}\right) -\nu _{K}^{rr}\left( A_{K-1}\right) \right] \end{aligned}$$

where $\nu _{K}\left( R^{0}\right)$ is defined as

48 $$\begin{aligned} \nu _{K}^{rr}\left( R^{0}\right) :=\mu \left[ A_{K-1}^{2}-\frac{r_{K-1}^{2}\left( A_{K-1}\right) }{\gamma _{K}^{2}}\right] \log \left[ r_{K}^{2}\left( R^{0}\right) \right] -\frac{1}{2}\mu \left( R^{0}\right) ^{2}\log \left[ \frac{r_{K}^{2}\left( R^{0}\right) }{\left( R^{0}\right) ^{2}}\right] . \end{aligned}$$

We have seen in (45) how the circumferential stress $T^{\theta \theta }$ relates to the radial stress $T^{RR}$. The average over that expression is

49 $$\begin{aligned} \overline{T_{K}^{\theta \theta }}=\overline{T_{K}^{RR}}+\overline{\kappa _{K}}. \end{aligned}$$

We have presented an expression for $\kappa _{K}$ in (46). The average over $\kappa _{K}$ is

50 $$\begin{aligned} \overline{\kappa _{K}}&=\frac{2\mu \left[ r_{K}^{2}\left( A_{K}\right) -\gamma _{K}^{2}A_{K}^{2}\right] }{\varDelta ^{2}\gamma _{K}{}^{2}}\log \left[ \frac{A_{K}^{2}r_{K}^{2}\left( A_{K}\right) }{A_{K-1}^{2}r_{K-1}^{2}\left( A_{K-1}\right) }\right] . \end{aligned}$$

According to (49), the expression for $\overline{T_{K}^{\theta \theta }}$ is the sum of $\overline{\kappa _{K}}$ (see 50) and $\overline{T_{K}^{RR}}$ (see 47). The average radial and circumferential stress components are depicted as horizontal lines in the respective layers in Fig. 10a (radial) and Fig. 10b (circumferential).

### Generating a Homeostatic State from a Prescribed Growth Profile.

The discretization and averaging process described above enables for a concise framework for studying growth dynamics. As the homeostatic state is defined by a growth profile—a function that is only constrained to be positive—a generic classification of dynamic behavior is likely untractable. Our intent, rather, is to briefly investigate stability and the rate of convergence in terms of the number of layers. For this, we restrict attention to a linear homeostatic growth profile $\gamma ^{*}\left( R^{0}\right)$, characterized by a single parameter, $C_{1}$,

51 $$\begin{aligned} \gamma ^{*}\left( R^{0}\right) =1+C_{1}\left( R^{0}-A_{0}\right) ,\qquad C_{1}\left( A_{N}-A_{0}\right) <1. \end{aligned}$$

Note that this growth profile satisfies $\gamma ^{*}\left( A_{0}\right) =1$, i.e., no growth at the inner boundary.

We obtain the discrete homeostatic stress profile $\left\{ \gamma _{i}^{*}\right\}$ from the continuous profile $\gamma ^{*}\left( R^{0}\right)$ by computing the average according to (36). The homeostatic stress ${\mathbf {T}}\left( \varvec{\gamma }^{*}\right)$ is computed from the discrete homeostatic stress profile $\left\{ \gamma _{i}^{*}\right\}$ according to (44) and (45). The homeostatic values $\overline{{\mathbf {T}}}\left( \varvec{\gamma }^{*}\right)$ are obtained as averages according to (47) and (49). It is important to note that the homeostatic stress is generated by prescribing a growth profile (51), which by definition ensures that the homeostatic stress is admissible.

### Growth Dynamics

We consider a growth law that generalizes (33) to *N* layers. The main difference with (33) is that the values for homeostatic stress are obtained by the linear growth profile.

The growth law reads

52 $$\begin{aligned}&{\dot{\gamma }}_{K}=\gamma _{K}\left\{ {\tilde{K}}\left[ \overline{T_{K}^{RR}}\left( \varvec{\gamma }\right) -\overline{T_{K}^{RR}}\left( \varvec{\gamma }^{*}\right) \right] +\overline{T_{K}^{\theta \theta }}\left( \varvec{\gamma }\right) -\overline{T_{K}^{\theta \theta }}\left( \varvec{\gamma }^{*}\right) \right\} , \nonumber \\&\qquad K=1\ldots N. \end{aligned}$$

In order to consider the stability of (52) in the neighborhood of the homeostatic state, we expand growth around its equilibrium values:

53 $$\begin{aligned} \gamma _{K}=\gamma _{K}^{*}+\varepsilon {\tilde{\gamma }}_{K}+{\mathcal {O}}\left( \varepsilon ^{2}\right) ,\qquad K=1,\ldots ,N. \end{aligned}$$

To linear order in $\varepsilon$, the dynamical system simplifies to

54 $$\begin{aligned} \dot{\tilde{\varvec{\gamma }}}={\mathbf {J}}\tilde{\varvec{\gamma }}. \end{aligned}$$

The eigenvalues of the Jacobian matrix ${\mathbf {J}}$ characterize the stability of (52) near the homeostatic state. The components of the $N\times N$ matrix ${\mathbf {J}}$ are

55 $$\begin{aligned} J_{ij}&=\left[ \gamma _{i}\left( {\tilde{K}}\frac{\partial \overline{T_{i}^{RR}}\left( \varvec{\gamma }\right) }{\partial \gamma _{j}}+\frac{\partial \overline{T_{i}^{\theta \theta }}\left( \varvec{\gamma }\right) }{\partial \gamma _{j}}\right) \right] _{\varvec{\gamma }=\varvec{\gamma }^{*}},\quad i,j=1,\ldots ,N. \end{aligned}$$

We characterize the stability in the neighborhood of the homeostatic state as a function of two non-dimensional parameters: The mechanical feedback anisotropy parameter ${\tilde{K}}$ and the slope of the homeostatic growth profile $C_{1}$. The latter appears in (55) through $\varvec{\gamma }^{*}$ (see Sect. 4.3).

**Fig. 11:**
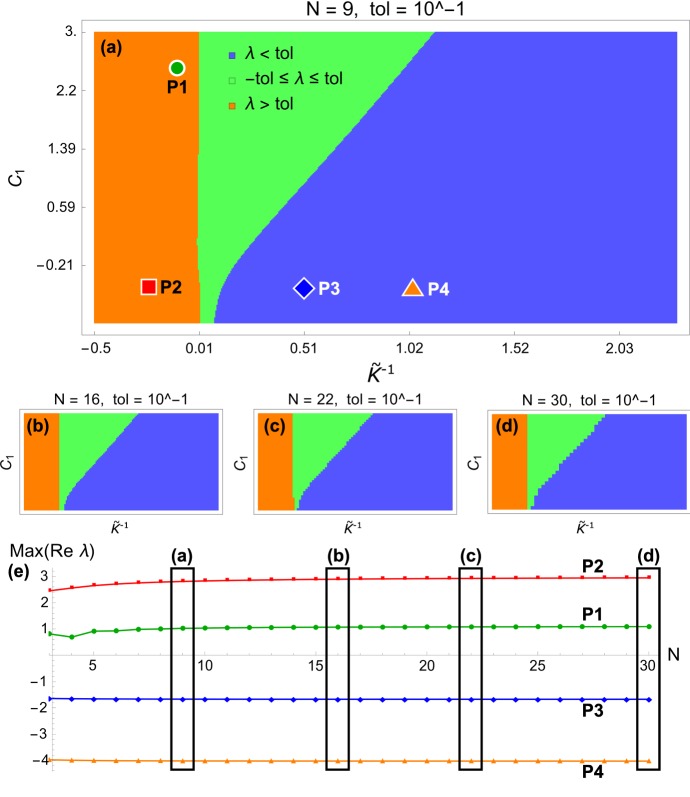
Bifurcation diagram and convergence for *N*-layered cylinder system. **a**–**d** The unstable (orange) and stable (blue) regions retain their shape for increasing values of *N*. **e** For a representative sample of points P1 to P4, the convergence of the largest eigenvalue is very good (see interpretation in text). The $\left( {\tilde{K}}^{-1},C_{1}\right)$ coordinates are $\text {P1}\left( 0.1,2.5\right)$, $\text {P2}\left( -0.25,-0.5\right)$, $\text {P3}\left( 0.5,-0.5\right)$, $\text {P4}\left( 1,-0.5\right)$. Other parameters are $\mu _{1}=1$, $A_{0}=1$, $A_{N}=2$

**Fig. 12:**
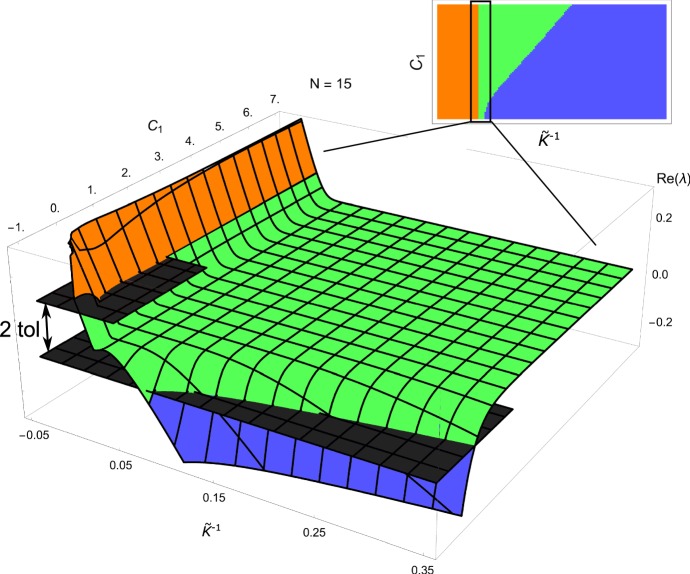
Detailed depiction of the shallow (green) region from Fig. 11. The large shallow region has a fine structure which is an artifact of discretization and is not expected in the full inhomogeneous system. For this reason, we choose a three color system in Fig. 11a–d, in which the shallow region and its fine structure are merged into one region defined by $-\text {tol}\le \lambda \le \text {tol}$. The two planes serving as upper and lower bounds of this region are depicted in dark gray. Values above $\lambda =\text {tol}$ are stable, below $\lambda =-\text {tol}$ are unstable

Figure 11a shows a bifurcation diagram of the stability of the dynamical system (52) as a function of ${\tilde{K}}^{-1}$ and $C_{1}$ for $N=9$ layers (note that unlike in Fig. 5, here we use the inverse of ${\tilde{K}}$ to focus on large circumferential stress). The regions are colored according to the largest real part of the eigenvalues $\lambda _{i}$ of ${\mathbf {J}}$, that is $\lambda =\text {Max(Re}\,\lambda _{1}, \text {Re}\,\lambda _{2}, \ldots \text {Re}\,\lambda _{N})$. There are three parameter regions: an unstable region (orange), a stable region (blue), and an undecidable region (green) for which $\lambda$ is within a small tolerance of zero. This last region is included as it is typically within numerical error and its inclusion allows to make precise statements about stability. This relatively shallow region of $\lambda$ is further explored in Fig. 12 and allows us to identify the clearly stable and clearly unstable regions of the diagram. Figure 11b–e shows that for increasing values of *N* (that is, a refinement of the discretization), the regions are practically unchanged (b–d), and that the largest eigenvalue of four selected points converges reliably to a finite positive (P1 and P2) or negative (P3 and P4) eigenvalue.

Thus, we see that there exist a region of stability, and a region of instability, which both persist (for large enough *N*) independently of the discretization. A strongly anisotropic growth law (${\tilde{K}}^{-1}$ close to zero or negative) is required for the system to be unstable. We also considered the convergence as *N* increases for a representative sample of points in the stable and unstable regions and confirmed that there was no significant change in $\lambda$. We expect that the stable and unstable regions represent the true behavior of the full (inhomogeneous) system discussed in Sect. 2. The intermediate (green) shallow region of eigenvalues has a more complicated structure due to the discretization that is not expected in the full system.

### Solid Nucleus Versus Pressurized Cylinder

Our choice for boundary conditions has been a solid nucleus at the inner wall of the cylinder, $r(A_0)=A_0$, and a no-pressure condition at the outer wall, $T^{RR}(B_0)=0$. These conditions are advantageous for mathematical analysis because the deformation map (8) can be easily integrated for a piecewise constant growth profile, with the result (39). Similarly, the radial stress (15) and circumferential stress (16) can be explicitly integrated for a piecewise constant growth profile, with stresses (44) and (45), respectively, and average stresses (47) and (49), respectively. Explicit forms of the average stresses are very advantageous for our model, in which derivatives of these expressions must be computed to obtain the Jacobian (55). In many biological tubular systems, however, the boundary condition will be a pressure difference between inner and outer cylinder walls, due to hydrostatic pressure maintained inside the tube. Consider the following boundary conditions:

56 $$\begin{aligned} T^{RR}\left( A_0\right) =P,\qquad T^{RR}\left( B_0\right) =0, \end{aligned}$$

where *P* is the prescribed pressure. Now, instead of (8), the integration of the radial map reads

57 $$\begin{aligned} r=\sqrt{a^{2}+2\int _{A_0}^{R^{0}}\gamma ^{r}({\tilde{R}})\gamma ^{\theta }({\tilde{R}}){\tilde{R}}\mathrm {d}{\tilde{R}}}. \end{aligned}$$

Notice that where previously we had the known nucleus position $A_0$, we now have the unknown current inner cylinder coordinate *a*. Therefore, even for a piecewise constant growth profile, (57) cannot be directly obtained because *a* is unknown. To determine *a*, it is necessary to integrate the radial stress $T^{RR}$ while using the boundary condition (56) in the form $-P=T^{RR}(B_0)-T^{RR}(A_0)$. This requires a numerical root finding algorithm (e.g., Newton’s Method) to determine *a*, as shown in Ben Amar and Goriely (2005) for a spherical shell. As the difference between a solid nucleus boundary condition and a pressure difference boundary condition (56) lies only in a difference for the inner cylinder wall, we expect that the techniques outlined in this article (stress-averaging, *N*-layered cylinder) will apply to the pressurized cylinder setup, albeit at extra computational cost.

## Conclusion

It is now well appreciated that growth can induce mechanical instabilities (Goriely and Ben Amar 2005; Ben Amar and Goriely 2005). The related problem that we have considered in this paper is the stability of a grown state through its slow-growth evolution. The question is not therefore about mechanical instability but about the dynamic stability of a preferred homeostatic state. While the former is characterized by a bifurcation from a base geometry to a more complex buckled geometry, occurring on a fast elastic timescale, the latter involves the system evolving away from a given stress state on the slow growth timescale. In general the homeostatic state is not homogeneous; hence, the issue of stability requires the analysis of partial differential equations defined on multiple configurations with free boundaries. There are no standard mathematical tools available to study this problem even for simple non-homogeneous systems. An alternative is to consider the stability of states that are piecewise homogeneous (in space). The problem is then to establish the stability of coupled ordinary differential equations describing locally homogenous states through the traditional methods of dynamical systems. Within this framework, we considered two relatively simple problems.

First, we considered the dynamical stability of a two-layer tube with different, but constant, growth tensors in each layer. We characterized the dynamics of the full nonlinear system and showed that the number of equilibria and their stability varies greatly and gives rise to highly intricate dynamics which we organized via several bifurcations. We identified a parameter region where the system is stable. We found that the growth dynamics of tubular structures in the neighborhood of the homeostatic equilibrium depends in a nontrivial way on the anisotropy of the growth response and that the equilibrium becomes unstable for highly anisotropic growth laws. This complexity of dynamics naturally raises the question about stability of homeostatic equilibria for more general systems.

Second, we showed that given a continuous law in a cylindrical geometry, we can introduce a suitable discretization of the problem that keeps all the characteristics of the continuous problem. We showed that for a linear growth law, there are clear regions where stability and instability persist independently of the discretization (for sufficiently large *N*). We expect that these regions represent the true behavior of the full inhomogeneous system. This result allows us to characterize the stability of a morphoelastic growing cylinder.

As our results (in particular Fig. 11) show, admissible homeostatic states can lead to either mechanically stable or unstable equilibria. This suggests a way to distinguish between physiological (stable) and pathological unbounded (unstable) growth. Indeed, our model also suggests a natural growth termination mechanism. The question of growth termination (what triggers a tissue to stop growing?) is a much-debated question in developmental biology. It has been particularly well studied in the model system of the *Drosophila melanogaster* wing disk where the morphogen Decapentaplegic (Dpp) has been identified as the main regulator of growth. A number of models propose growth regulation and termination based on a combination of mechanical effects and Dpp concentration. Some of them are continuum models (Aegerter-Wilmsen et al. 2007; Ambrosi et al. 2015), others are vertex models (Shraiman 2005; Hufnagel et al. 2007) but none of them is entirely satisfactory (Vollmer et al. 2017). We hope that the dynamical stability of homeostatic states offers an alternative way of looking at growth termination, which emerges naturally in our model as a stable equilibrium.

While we have only scratched the surface of the complex dynamic behavior that exists in such systems, the framework presented here provides a tool to explore growth dynamics and stability of homeostatic states and finally address some of the fundamental challenges of morphoelasticity (Goriely 2017): What growth laws, in general, would lead to dynamically stable homeostatic states? What is the final size of a growing organism for a given growth law? What are the conditions under which growth dynamics produces oscillatory growth?
